# Engineering-geological scientific study investigating the relationship of slope gradients to two basic types of rock masses in the Czech Republic

**DOI:** 10.1038/s41598-026-51444-8

**Published:** 2026-05-18

**Authors:** Marian Marschalko, Dariusz Popielarczyk, Kateřina Růžičková, Jan Růžička, Dominik Niemiec, Radek Pohanka

**Affiliations:** 1 Department of Geological Engineering, Faculty of Mining and Geology, VŠB-Technical University of Ostrava, 708 33 Ostrava, Czech Republic; 2 Department of Geodesy, Faculty of Geoengineering, University of Warmia and Mazury in Olsztyn, 10 719 Olsztyn, Poland; 3 Department of Geoinformatics, Faculty of Mining and Geology, VŠB-Technical University of Ostrava, 708 33 Ostrava, Czech Republic

**Keywords:** Slope gradients, Soil and rock mass, Engineering geological zones, DMR 4G, Statistical characteristics and geospatial analysis, Average slope gradient, 25/50/75 Quantile, Environmental sciences, Solid Earth sciences, Engineering

## Abstract

The presented scientific study evaluates and quantifies the relationship of slope gradients to two fundamental types of rock masses in the Czech Republic. Investigating slope gradients within engineering geological zones of soil or rock and weathered rock masses (these areas were analyzed using a 1:50,000-scale map, whereas a previously published study used only a 1:500,000-scale map) is a fundamental scientific inquiry, as it compares the behaviour of these two basic mass types in engineering geology, geotechnics and geospatial analysis based on slope gradient values. Slope gradient is essentially the sole parameter observable on the surface across the entire country, allowing us to compare the behaviour of these two mass types. The study was divided into two parts, with the first study evaluating a group of engineering geological zones with Quaternary and pre-Quaternary geological structures (Study 1). The second study, which is of greater scientific significance (Study 2) assessed two groups of engineering geological zones with rocks and weathered rocks and their eluvium (rocks and weathered rocks), and zones with soil engineering geological characteristics. For all variations and zones, statistical characteristics were determined (average slope gradient, 25% quantile, 50% quantile, 75% quantile, and maximum slope gradient). It was found that the groups exhibit significant statistical differences in slope gradients in the Czech Republic. The most significant finding from Study 2 was that the group of engineering geological zones with rocks and weathered rocks and their eluvium (Group 2A) and the group of soil zones (Group 2B) had a difference between the minimums of average slope gradients of 3.0°, representing a 41% share of the total differences. In contrast, the difference between the maximums of average slope gradients in both groups of masses was even greater at 4.4°, representing a 59% share. When comparing the difference between the minimum and maximum of average slope gradients in the first (2A) and second group (2B), a difference of 5.6° (57% share of the total differences) was found in the first group, whereas in the second group, it was lower at 4.2° (43%). It is evident that soil masses and masses of rocks and weathered rocks must manifest differently due to primarily distinct physical–mechanical properties. This is logically reflected in slope gradients, as demonstrated and quantified in the Czech Republic through this study. Soil masses have lower slope gradients compared to masses of rocks and weathered rocks with their eluvium, which is entirely logical and corresponds to their material nature and the structure of the rock mass, reflecting in physical–mechanical properties observed on the surface in a single parameter, namely the assessed slope gradient.

## Introduction

***The subject*** of the presented ***scientific study*** is the assessment of slope gradients in relation to engineering geological zones in the Czech Republic. Engineering geological zones are areas where we encounter similar engineering geological and ground conditions. This implies that areas defined in this way (polygons) in engineering geology and geotechnics are scientifically the most relevant for slope gradient studies.

The research builds on a previous scientific study^1^, with the newly presented scientific study featuring several significant differences and novelties compared to the previous one. The first major difference is that the newly presented study has ten times greater accuracy in the interest area of the Czech Republic. While the previous study was conducted at a scale of 1:500,000, the new study is at a scale of 1:50,000, representing a tenfold increase in accuracy. The second, even more crucial difference and novelty is that the previous study categorized engineering geological zones only into Quaternary and pre-Quaternary and did not address the differences in engineering geological environments from the perspective of rock masses, which are essential for engineering geological assessment. This study classifies rock masses into Rock and Weathered Rock Mass and Soil Mass. This is a fundamental novelty of this article in relation to slope gradients in a specific country and to the two basic types of masses. The newly adopted approach is absolutely essential in terms of the continuity of previous research. The original research considered only a stratigraphic approach, which is not crucial from the perspective of engineering geology and the physical–mechanical properties. The new approach is entirely engineering-geological, as the distinction between soils, soft rocks (semisolid rock) and hard rocks (solid rock) is fundamental from the standpoint of engineering geology and the physical–mechanical properties of the geological environment. The newly used methodology and evaluation are universal from the perspective of evaluating other countries or smaller regions.

***Slope gradient*** is one of the most important characteristics that we have on Earth. It is one of the few parameters that we can observe anywhere on the Earth’s surface. The value of slope gradient is conditioned by a range of properties, whether they belong to rocks with soil characteristics^2^ or to rocks and weathered rocks^3,4^. Another influencing factor^5^ is the genetic character and geological structure, as well as the nature and intensity of geodynamic processes, and more.

Slope gradient^6^ is ***a boundary condition and a factor*** in the foundation engineering of all structures. Due to this relationship, it is of interest to scientific disciplines such as engineering geology, geotechnics, and others. Slope gradient influences the two most crucial parameters in structure foundation, namely bearing capacity^7–10^ and settlement^11–13^.

***The investigation of slope gradients*** is a part of all engineering geological investigations^14^ for various types of structures. It is also part of monitoring specific purposes identified during geotechnical monitoring^15,16^.

There are other scientific disciplines where slope gradient is a key parameter. One such discipline is geomorphology^17–19^. Slopes can have natural or anthropogenic characteristics^20–22^.

Slope gradient determines the stability of slopes^23,19^, making it a part of hazard assessment^24^. When slope stability is compromised, landslides occur^25–28^. Various methods, such as remote sensing^29^, mapping, geophysical methods, technical exploration, field geotechnical tests, geotechnical monitoring^30,6^, and others, are employed to study landslides. In addition to slope gradient, landslides are influenced by factors such as earthquakes^31–34^, precipitation, other climatic conditions, anthropogenic factors, geodynamic processes, and many more.

There are several important mapping datasets where slope gradient is part of the assessment criteria. One such dataset is engineering geological maps^35^. A powerful tool for regional landslide assessment is landslides susceptibility assessment^36^, where slope gradients are a crucial parameter in this assessment process.

## Scientific aim of the study

**Fig. 1:**
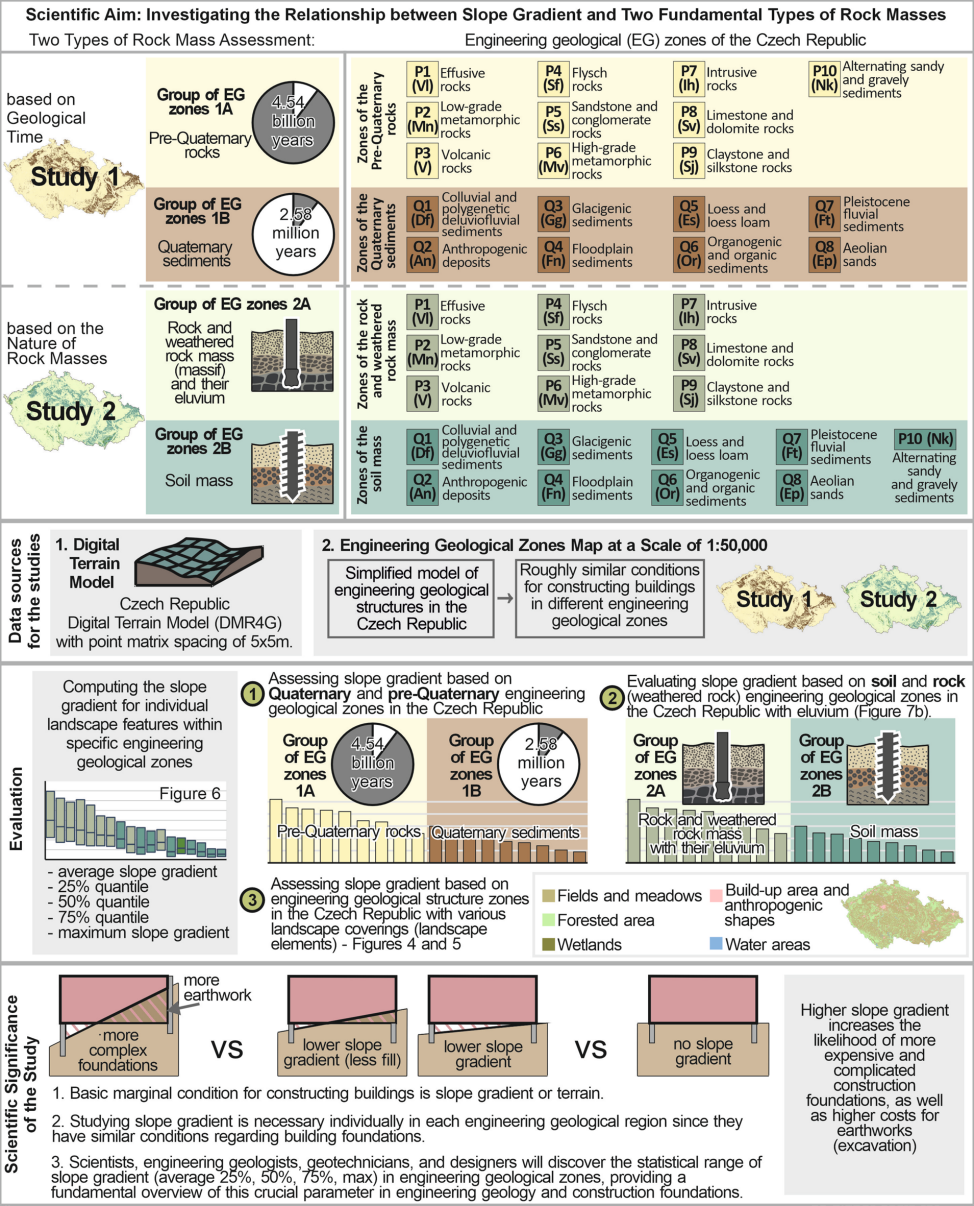
Schematic representation of the scientific objectives of the study.

The engineering geological study investigates the relationship of slope gradients (Fig. 1) to two fundamental types of rock masses in the Czech Republic. The scientific research was conducted in two variants of scientific studies. The first study (***Study 1***) assessed slope gradients in relation to two groups of zones based on ***geological time***. These groups included pre-Quaternary (***Group of Engineering Geological EG Zones 1A***) and Quaternary engineering geological zones (***Group of EG Zones 1B***). The temporal assessment of engineering geological zones is fundamentally related to the ***character of the rock mass types.*** The second study (***Study 2***) evaluated slope gradients in relation to two groups of zones based on the character of the rock mass, which is crucial for engineering geology and geotechnics. These groups were engineering geological zones of rock and weathered rock masses with their eluvium (***Group of EG Zones 2A***) and soil engineering geological zones (***Group of EG Zones 2B***). In this study, we used the delineation of engineering-geological zones prepared by the authors of this paper within earlier projects^37^. These zones were secondarily derived from the 1:50,000 geological map^38^.

The only difference between the first and second studies was the engineering geological zone “Alternating sandy and gravely sediments (Nk).” In the first study, this zone was part of the pre-Quaternary zones (Group 1A), all of which, except for this zone, have characteristics of rock and weathered rock formations. In the second study, it was relocated to the soil engineering geological zone group (Group 2B), where only Quaternar engineering geological zones are found. The reason was simple: unlike other pre-Quaternary zones, this zone has soil characteristics, while others have characteristics of rock and weathered rock formations.

The research was processed based on ***two fundamental datasets***, the first being the ***digital terrain model*** of the Czech Republic, and the second being the ***engineering geological map of zones*** at a scale of 1:50,000, depicting areas (zones) with an integrated view of similar engineering geological structures and similar conditions regarding construction foundations.

Within ***the study***, the calculation of slope gradients in individual engineering geological zones from the digital terrain model of the Czech Republic (DMR4G—Digital Terrain Model) with point matrices spaced at 5 × 5 m intervals was ***carried out***. The digital terrain model had a central error of 1 m in forested areas. In preprocessing, this point matrix representation was converted to a matrix of square cells with dimensions of 5 × 5 m. The slope gradient calculation itself took place by deriving the slope from the relief raster using a computation based on the analysis of the surroundings. The slope was calculated for the central cell using a convolutional floating window. For each cell, the surroundings of a 3 × 3 cell area were evaluated, and from these obtained 9 elevation data, the slope for the central cell was calculated. The calculation of individual statistical characteristics (average slope gradient, 25% quantile, 50% quantile (median), and 75% quantile of slope gradient, maximum slope gradient) was calculated cumulatively for all polygons with the same type of engineering geological zones. Thus, each individual type of zone has a series of polygons in the Czech Republic, but each statistical characteristic was calculated only once.

Within the conducted study, the calculation of slope gradients also took place within individual engineering geological zones in relation to the nature of landscape elements. The following landscape elements were considered: Fields and meadows, Forested area, Wetlands, Build-up area and anthropogenic shapes, Water areas.

***The scientific significance of the study*** lies in the fact that slope gradient, or terrain slope, is a fundamental boundary condition for construction foundations. Therefore, when founding any structure, the character and foundation method of the structure are significantly influenced by the slope gradient. For example, the geometry of building footprints often changes with increasing slope. Typically, it changes from a square footprint to a rectangular one because an increasing slope causes a larger volume of excavation and backfill work for a square footprint. However, it also causes deterioration of slope stability because the cut into the slope is longer in this case. At the same time, as the length of the excavation increases during foundation construction, the likelihood of poorer load-bearing capacity increases if the excavation alters the configuration of layer thicknesses beneath the structure from uniform thickness to varying thicknesses. Difficulties related to load-bearing capacity and settlement are often associated with variably thick layers of foundation soils beneath the foundation joint.

Consequently, slope gradient can change the values of bearing capacity and settlement. Therefore, understanding the values of slope gradient in individual types of geological structures is essential scientific and practical knowledge that influences all construction activities, both from the perspective of building foundations and earthworks^39,40^. A steeper slope implies greater costs and technical complexity for building foundations. A lower slope means a smaller amount of fill material (embankments), which can cause issues with uneven settlement and bearing capacity of structures. Conversely, flat terrain may mean simpler and cheaper options in terms of building foundations and earthworks, but caution must be exercised in selecting a location in terms of flooding, as minimal slope gradients are characteristic of ***floodplain*** areas.

Scientists, engineering geologists, geotechnicians, and designers will, based on the presented study, find the statistical range of slope gradients (average 25%, 50%, 75%, max) in engineering geological zones of the Czech Republic, gaining a basic overview of this significant parameter in engineering geology and construction foundations. A similar approach can then be utilized in other countries. The higher the slope gradient, the greater the likelihood of more expensive and complicated foundation construction, as well as more expensive earthworks (excavation).

**Fig. 2:**
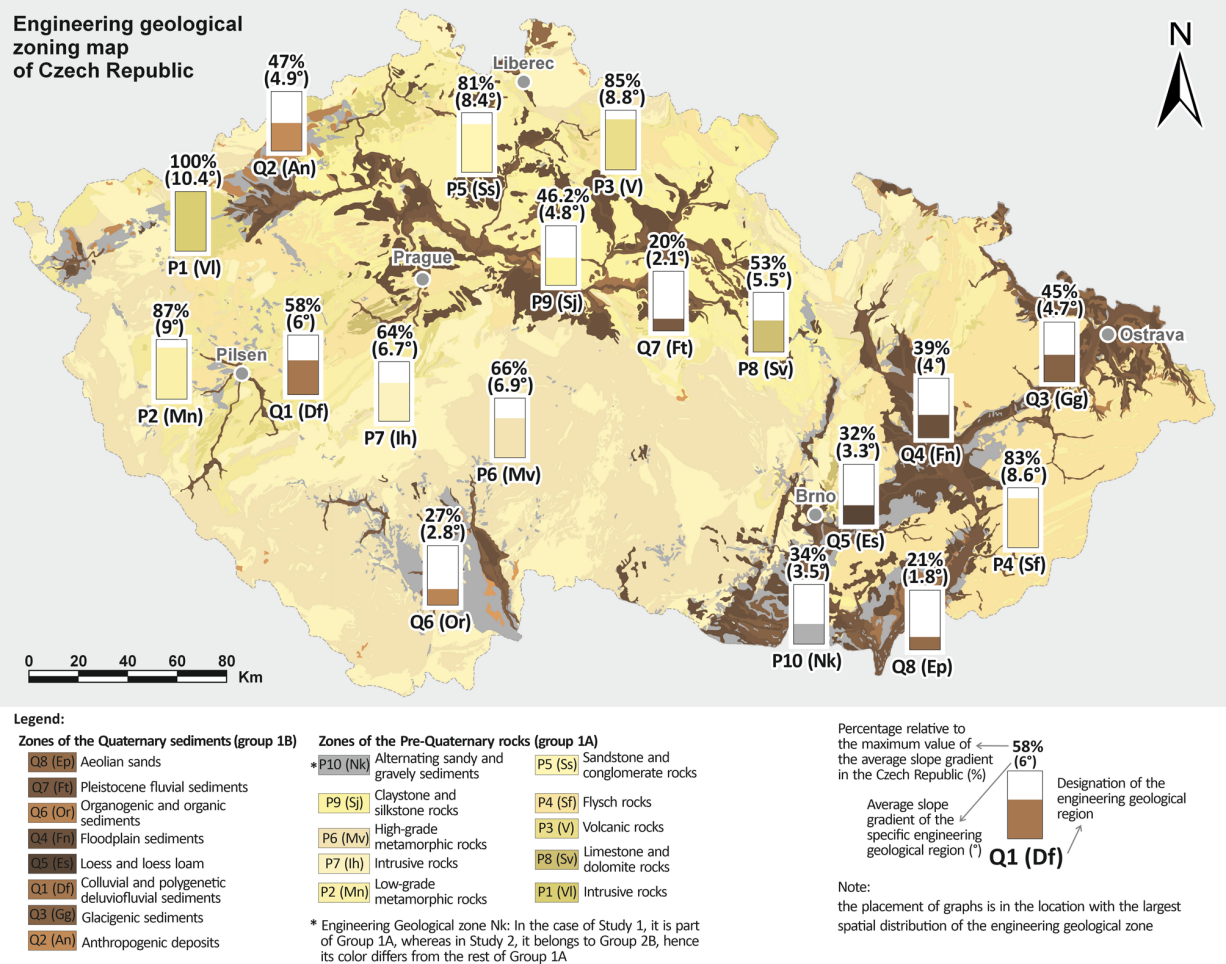
Schematic map of engineering geological zones in the Czech Republic based on a 1:50,000 scale (scale depicted graphically in the image) with indication of average slope gradients in individual engineering geological zones and representation of the percentage relative to the maximum average slope gradient in the Czech Republic.

The implemented study was conducted within the territory of the Czech Republic, focusing on the evaluated ***distribution of slope gradients*** in various ***engineering geological zones***^41,42,37^. The spatial constraints of individual zones were determined by a ***1:50,000*** map (Fig. 2). In the Czech Republic, two predominant groups of engineering geological zones exist.

***The first group,*** with a higher spatial representation, is comprised of pre-Quaternary engineering geological zones formed by foundation soils of rocks and weathered rocks with their eluvium. This group has a widespread representation across the Czech Republic, with its highest extent in the southern and southwestern parts. ***The second group***, with a lower spatial representation, consists of Quaternary engineering geological zones formed by soil-based foundation soils. This group has the greatest representation in the eastern part of the country, associated with the Carpathian frontal depression, and also in the northern part, with spatial limitations imposed by the Czech Cretaceous Plateau.

It is apparent that the first group exhibits higher average slope gradients (Fig. 2), as depicted in the Fig., including the value of the percentage relative to the maximum recorded average slope gradient in the Czech Republic. This value (10.4°) was recorded in the engineering geological zone of intrusive rocks (Vl).

## Visualization of real slope gradients in engineering geological zones within assessed statistical characteristics

**Fig. 3:**
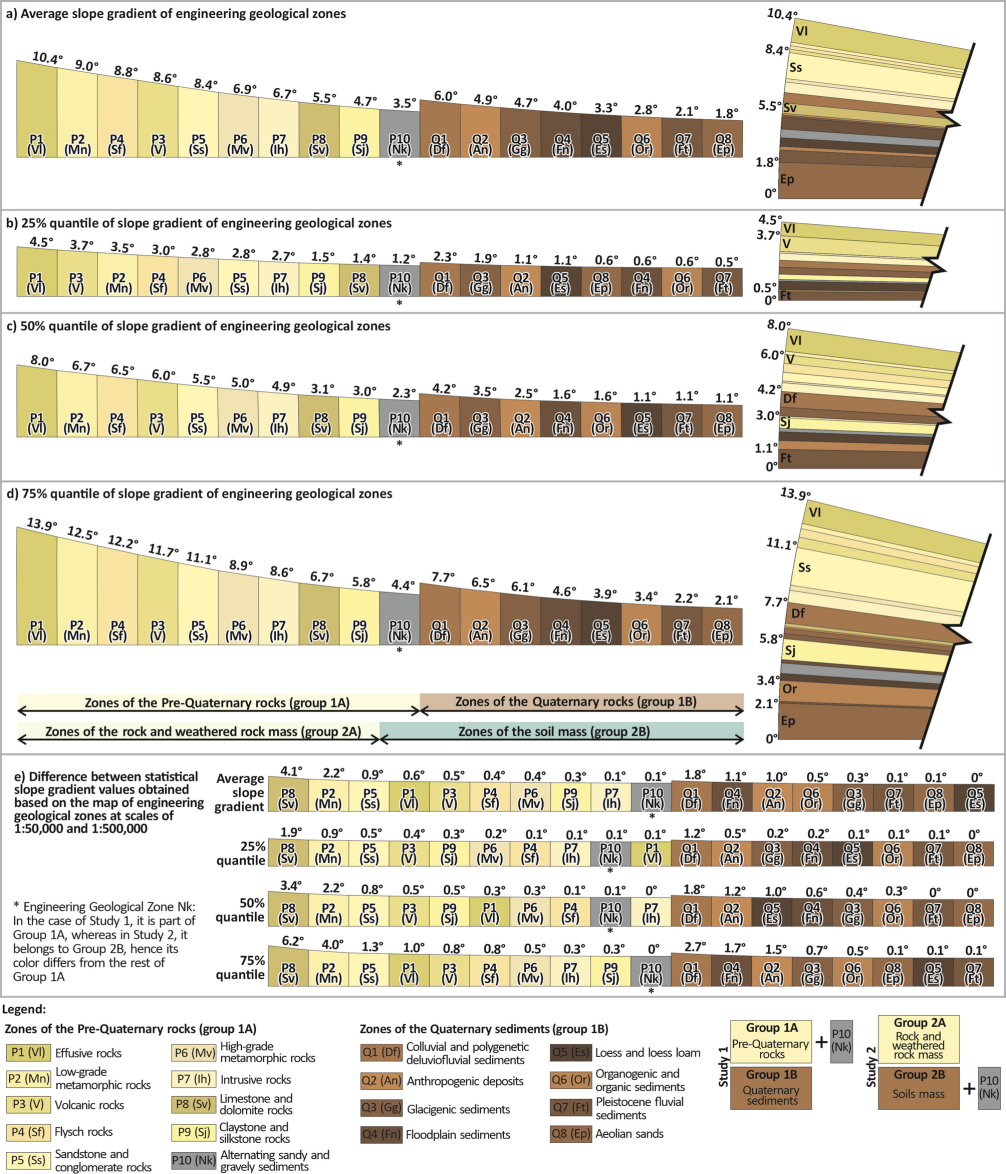
Visualization of real slope gradients in engineering geological zones within assessed statistical characteristics, (**a**) Average Slope Gradient of Engineering Geological Zones, (**b**) 25% Quantile, (**c**) 50% Quantile (Median), (**d**) 75% Quantile.

In Fig. 3, ***real*** and unaltered ***slope gradients*** are depicted. Thus, no different horizontal and vertical scales are applied here. The reason for this is the necessity of presenting an authentic representation, allowing for an undistorted view of slope gradients.

When evaluating the individual statistical characteristics of slope gradients, the first evaluated characteristic is the ***average slope gradient*** (Fig. 3a). Here, ***the first group*** of pre-quartile engineering geological zones (***group EG zones 1A***—see Fig. 1) in study 1 ranges from a minimum value of 3.5° to a maximum value of 10.4°. Whereas a similar group of rock and weathered rock zones (***group EG zones 2A***—see Fig. 1) in study 2 ranges from a minimum value of 4.7° to a maximum value of 10.4°. Therefore, the difference in this evaluation lies only in the minimum values, which represents 1.2°.

***The second evaluated group*** of quartile engineering geological zones (***group EG zones 1B***) ranges from a minimum value of 1.8° to a maximum value of 6.0°. A similar group in the soil mass variant (***group EG zones 2B***) reaches the same values because the engineering geological zone Nk Alternating sandy and gravely sediments is within the evaluated group, thus not reflected in the minimum and maximum values.

Regarding the second evaluated characteristic, the ***25% quantile*** (Fig. 3b) for ***the first evaluated group*** (group EG zones **1A**) of pre-quartile engineering geological zones has a minimum value of 1.2° and a maximum value of 4.5°. Compared to variant **2A** (zone of rocks and weathered rocks with their eluvium—group EG zones 2A), the difference lies only in the minimum value, which is 1.4°. ***Another group*** of engineering geological zones in variants **1B** and **2B** achieves analogous values. Thus, the minimum value is 0.5° and the maximum is 2.3°.

The third evaluated statistical characteristic, the ***50% quantile*** (Fig. 3c), differs in the variants of zones 1A and 2A, where the minimum value in variant 1A is 2.3° and in variant 2A is 3.0°. Whereas the maximum value is the same for both and reaches a value of 8.0°. Comparing with variants 1B and 2B, here, the slope gradients are lower (minima reach analogous values of 1.1° and maxima 4.2°).

The fourth evaluated statistical parameter in Fig. 3 is the ***75% quantile*** (Fig. 3d), which differs in minimum values between variant 1A and variant 2A, where the former reaches a value of 4.4° and the latter 5.8°. Conversely, the maximum value in both variants is the same, with a slope gradient of 13.9°. The variant of group zones 1B and 2B achieves lower values compared to the first group 1A and 1B, with the minimum value being the same for both at 2.1°. The maximum value of the slope gradient is also the same, at 7.7°. The observed trends in values indicate that soil masses have lower slopes compared to masses of rocks and weathered rocks with their eluvium. This proposition is inherently logical and aligns with the inherent ***material characteristics*** and the structure of the rock mass, which is reflected in the physical–mechanical properties manifested on the surface in a single parameter, namely the evaluated slope gradient.

In the past, a study of slope gradients was conducted based on a scale of 1:500,000^1^, whereas the current study is ten times more precise in terms of scale. The differences found in the statistical values are shown in Fig. 3e.

## Spatial distribution of landscape elements within engineering geological zones hierarchically arranged by average slope gradient

**Fig. 4:**
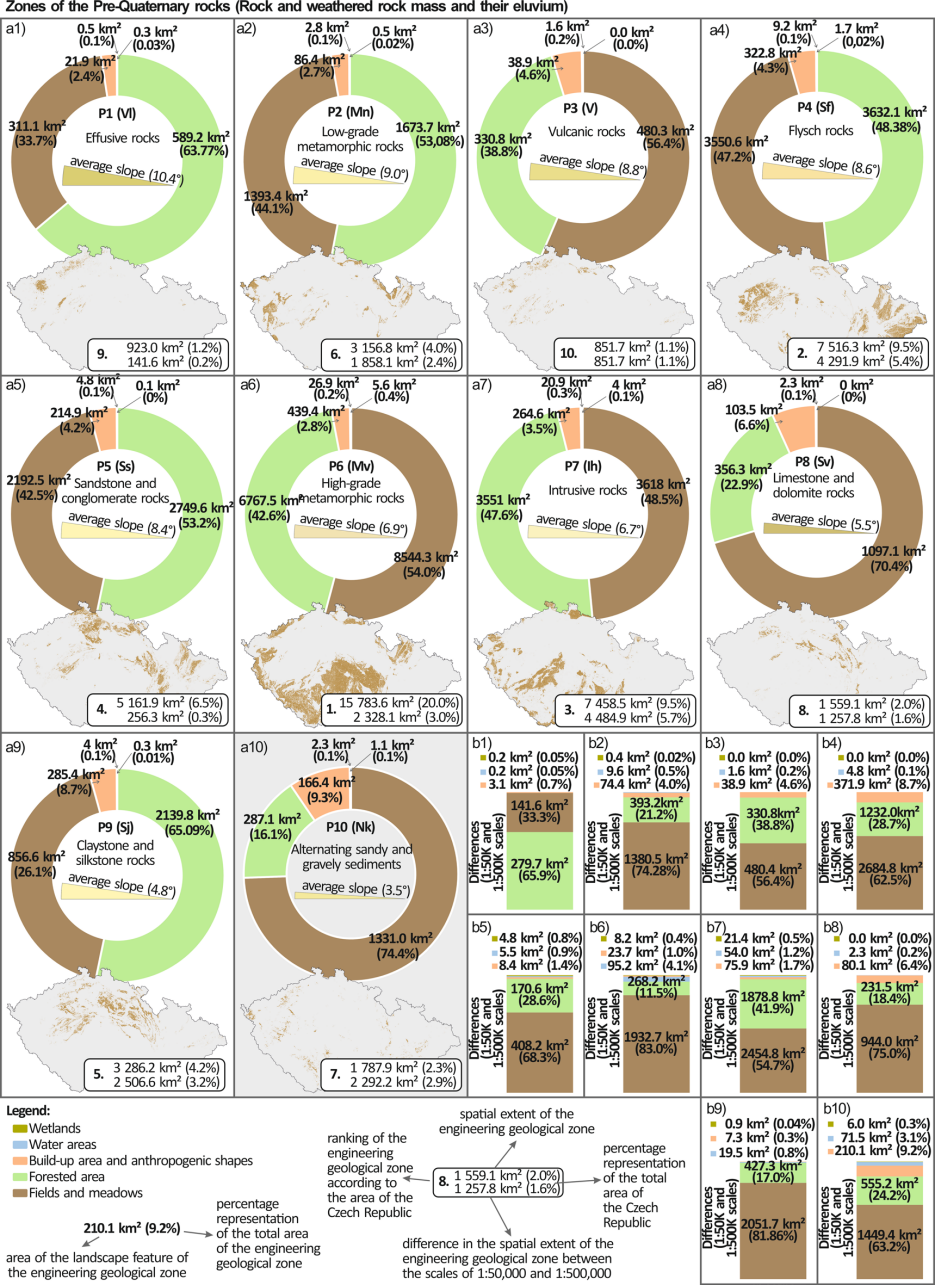
Spatial distribution of landscape features within individual pre-Quaternary engineering-geological zones of the Czech Republic (**a**) quantification and structure of landscape features in a group of pre-Quaternary engineering-geological zones arranged by increasing average slope gradient, (**b**) quantification of differences in the results of landscape features between map scales 1:50,000 (current study) and 1:500,000 (previous study).

**Fig. 5:**
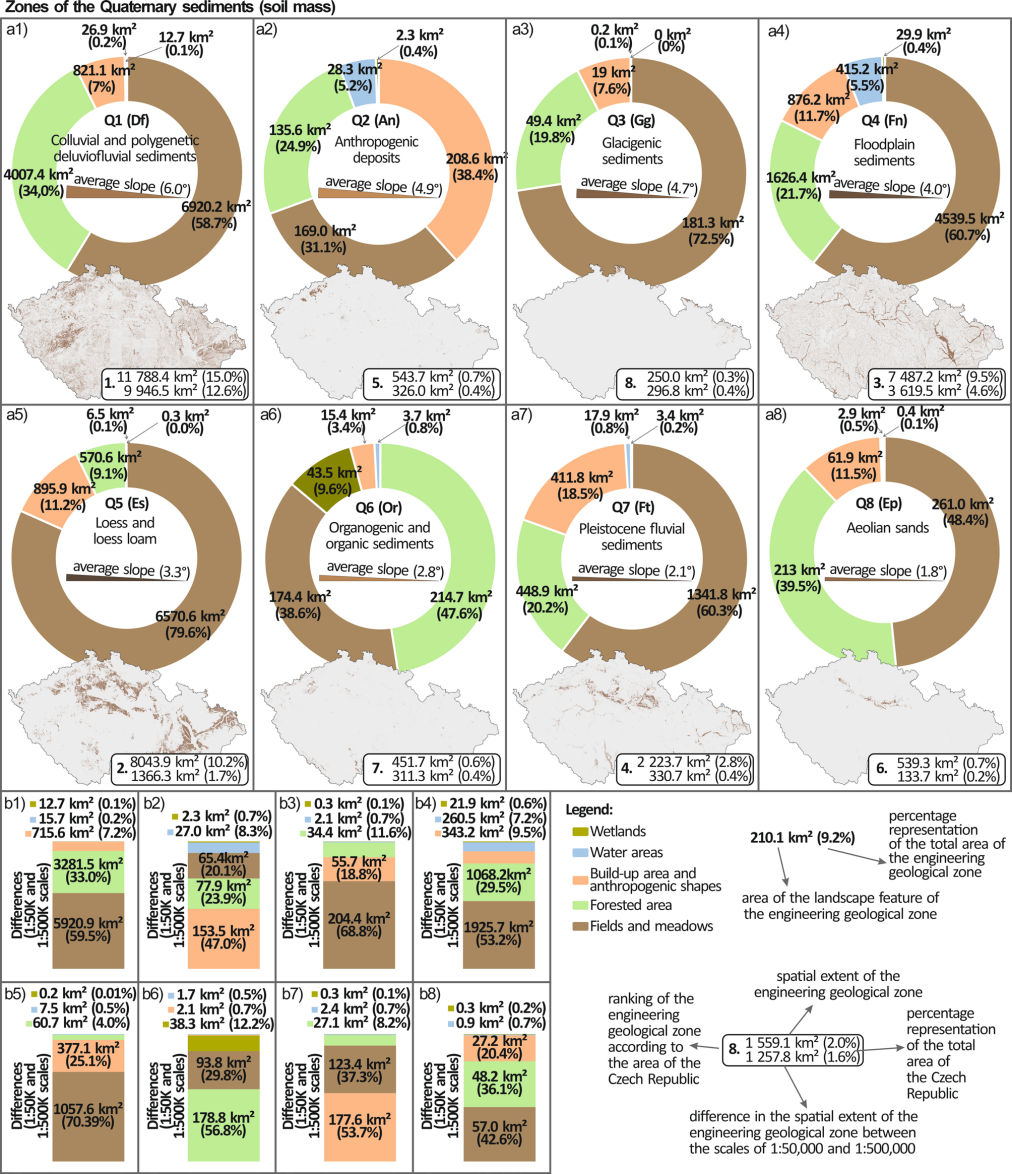
Spatial distribution of landscape features within individual Quaternary engineering-geological zones of the Czech Republic (**a**) quantification and structure of landscape features in a group of Quaternary engineering-geological zones arranged by increasing average slope gradient, (**b**) quantification of differences in the results of landscape features between map scales 1:50,000 (current study) and 1:500,000 (previous study).

In the research of slope gradients in engineering geological zones of the Czech Republic, the ***distribution of landscape elements*** was examined (Fig. 4, 5). Comparing the difference in the distribution of landscape elements in pre-quartile engineering geological zones (Fig. 4a, ***rocks and weathered rocks with their eluvium***) with higher slope gradients compared to quaternary zones (Fig. 5a, ***soil zones***) with lower slope gradients, it is apparent that the first group has a different distribution of landscape elements than the second.

In the first group, landscape elements fields and meadows and forested area completely dominate, while the landscape element build-up area and anthropogenic shapes is minor. Whereas the second group has a more diverse distribution of landscape elements. Here, there are zones with a distribution similar to the first group, but there are also zones where the distribution is different, with the landscape element build-up area playing a greater role. In contrast to the first group, the second group more prominently features the landscape element water areas and wetlands.

As part of the research, the difference between the results of the analysis obtained from the less precise engineering geological map of 1:500,000 (study conducted in the past) and the more precise map of 1:50,000 (study conducted within the presented article) was calculated, illustrating the differential area of landscape elements in Figs. 4b and 5b.

## Evaluation and comparison of the first and second study

**Fig. 6:**
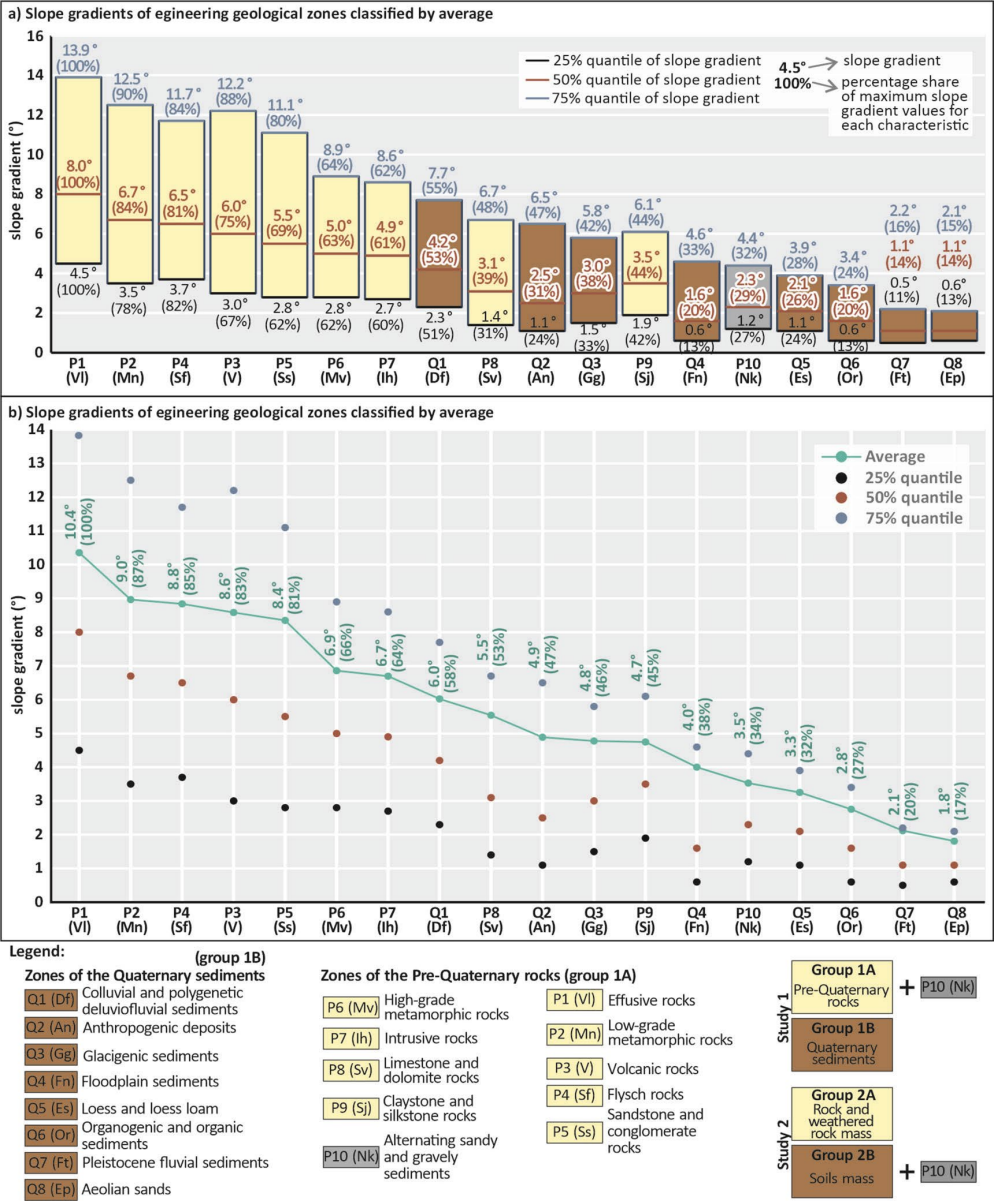
Slope gradient values in engineering geological zones of the Czech Republic for the first and second studies combined: (**a**) Box-plot graph (**b**) Combined line graph highlighting the average slope gradient with a scatter plot of statistical values—25% quantile, 50% quantile, 75% quantile.

When comparing the box plots of slope values in individual engineering-geological zones, it is evident that zones characterized by rock and weathered rock masses exhibit significantly wider ranges of box plots compared to zones with soil masses (Fig. 6a).

The evaluated study further demonstrated (Fig. 6a) that the range of values in the box plot gradually increases from zones with poorer physico-mechanical properties to zones with better physico-mechanical properties (direction of assessment graph from right to left). Another finding of the study is the fact that soil masses have a narrower range of box plot values for slope gradients compared to zones with rock and weathered rock masses with their eluvium.

When assessing the line chart (Fig. 6b), it is clear that the average value essentially linearly increases from engineering-geological zones with poorer physico-mechanical properties to zones with better physico-mechanical properties (direction of assessment graph from right to left). Consequently, the opposite statement also holds true, namely, that the line chart of average slope gradient values linearly decreases from zones with better physico-mechanical properties to zones with poorer physico-mechanical properties.

It is a well-known fact that soil masses have inferior physico-mechanical properties compared to rock and weathered rock masses. The study’s significant scientific value lies in demonstrating this commonly known fact through specific statistical values of slope gradients in the particular environment of engineering-geological zones in the Czech Republic.

An important scientific finding of the study is that all statistical characteristics of slope gradients in the compared groups of engineering geological zones exhibit analogous trends. There is only one exception, which is the maximum values. Here, the trend is partially different, leading to greater variability in slope gradients at their maximum values. From a descriptive standpoint, the most relevant statistical characteristic can be considered as the mean, also because other statistical characteristics show similar trends.

**Fig. 7:**
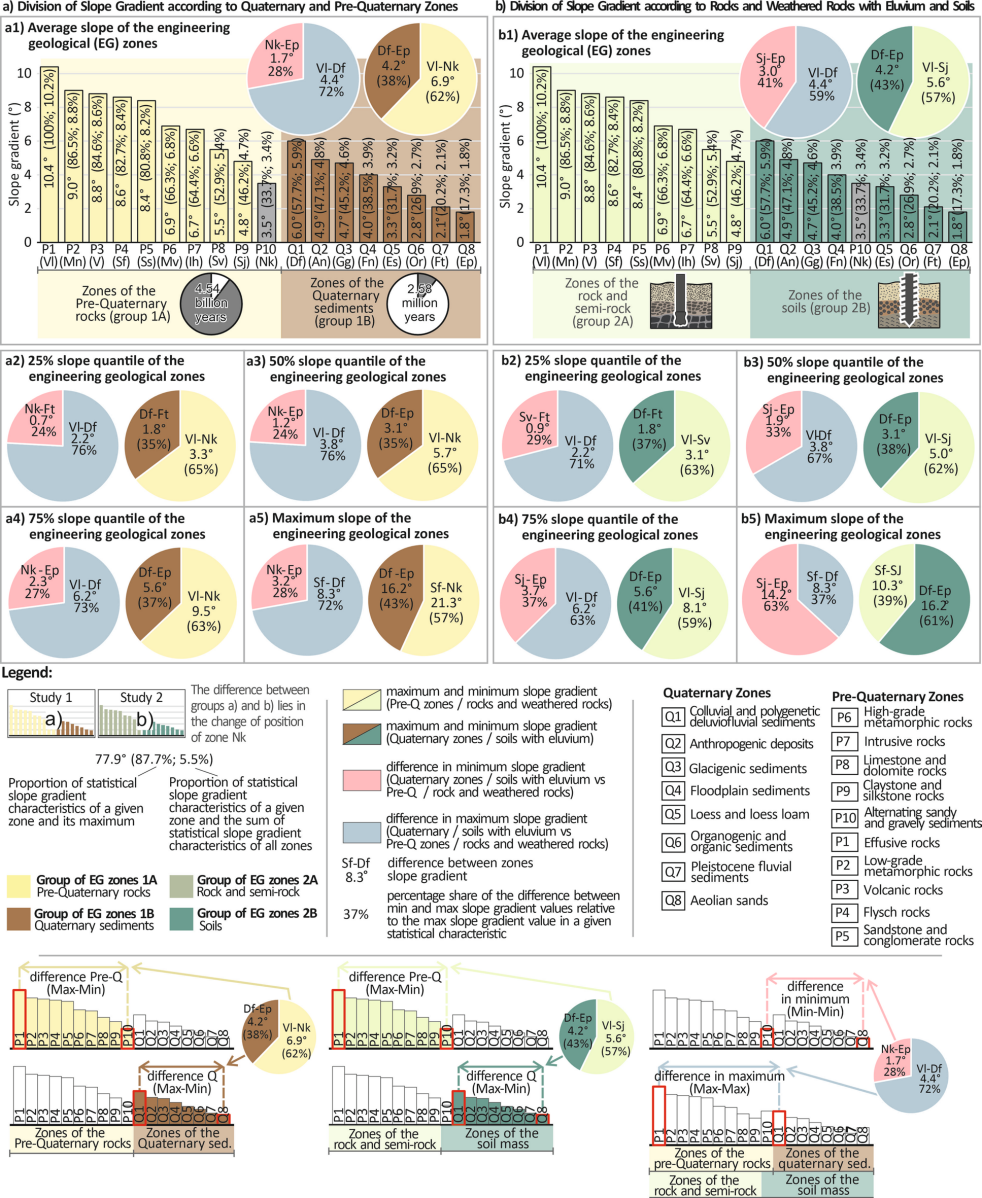
Quantification of slope gradients within study 1 (Comparison of slope gradient values in pre-quaternary and quaternary engineering geological zones) and within Study 2 (Comparison of slope gradient values rock and weathered rock with eluvium, and soil engineering geological zones); (**a**) Study 1 (a1—average slope gradient, a2—25% quantile, a3—50% quantile, a4—75% quantile, a5—maximum slope gradient); (**b**) Study 2 (b1—average slope gradient, b2—25% quantile, b3—50% quantile, b4—75% quantile, b5—maximum slope gradient).

A significant result of the study is the determination of ***differences in statistical values of slope gradients*** between two basic types of rock masses (Fig. 7a1, b1 to a5, b5—pie charts with explanations in the legend) from the perspective of engineering geology and geotechnics. Therefore, that the most important difference for comparison lies between masses of rock and weathered rock formations with eluvial cover (Group of engineering geological zones 2A) representing pre-Quaternary rocks with their eluvium (Group of engineering geological zones 1A without the Nk zone) and soil masses (Group of engineering geological zones 2B) representing Quaternary sediment areas (Group of engineering geological zones 1B with the Nk zone).

It is necessary to perceive the difference between the minimum and maximum values of slope gradients as the most representative for the statistical characteristic of ***average slope gradient*** in both groups 2A and 2B (study 2) (Fig. 7a1, b1). When comparing the differences in minimum and maximum slope gradients (difference between Group of EG zones 2A and Group of EG zones 2B), it is apparent that the difference in minimums represents 3.0° and the difference in maximums represents 4.4° (Fig. 7b1). Thus, the percentage difference in minimums accounts for 41% of the total sum of both differences, whereas the difference in maximums accounts for up to 59% of the total sum of both differences. Thus, the difference between groups is distinctly evident in the maximum values of slope gradients compared to the minimum values.

If we compare the group (study 1) of engineering geological zones with different chronostratigraphic periods (difference between Group of EG zones 1A—Quaternary, and Group of EG zones 1B—pre-Quaternary), the research showed that the difference in minimums is 1.7° (28%—proportion to the total sum of both differences) and 4.4° (28%).

When comparing the determined differences in both evaluated studies 1 and 2, it is evident that the evaluation system of engineering geological zones into Quaternary and pre-Quaternary (study 1—difference of 1.7°, 28%) brought greater differences in minimum values than the evaluation system of zones into soil and rock and weathered rock formations in study 2 (difference of 3.0°, 41%). Consequently, the percentage difference between minimums of both studies 1 and 2 is 13% (difference between 41 and 28%). The difference between studies 1 and 2 in maximum values is not present (4.4°) because the zone with maximum values is the same for both evaluated groups, thus there could not be a difference. However, the difference lies in the percentage representation, where in the case of study 1 (72%) and 2 (59%) (Fig. 7a1, b1).

When evaluating the values of all statistical characteristics (***average slope gradient, 25% quantile, 50% quantile, 75% quantile***) except for the maximum slope gradient, the difference in slope gradient values between both groups of engineering geological zones in studies 1 and 2 follows a similar trend in terms of ***mutual ratio*** (Fig. 7).

When comparing the differences in ***minimum and maximum values between groups*** of groups of engineering geological ***zones with pre-Quaternary*** geological structure (predominantly rock and weathered rock masses and their eluvia) and differences in ***minimum and maximum values*** of groups of engineering geological ***zones with Quaternary*** unconsolidated sediments (soil masses), an obvious trend is observed. The group of Quaternary soil zones 1B (brown sector of pie charts) reaches roughly one-third of the values (range of 35 to 38%—3.1° to 5.6° for more significant statistical characteristics—average slope, 50% quantile, and 75% quantile) compared to the second group 1A (yellow sector of pie charts).

When evaluating the ***differences between minimum values*** of both assessed groups (Fig. 7—pink sector of pie charts), it is apparent that this difference essentially represents one-fourth of the values compared to ***differences in maximum values*** (Fig. 7—light blue sector of pie charts). This trend is observable in all statistical characteristics (Fig. 7a1 to a5) except (partially only) in the statistical characteristic of maximum slope gradient values.

The highest recorded ***value of the percentage share*** between ***maximum values*** of evaluated zone groups (between zone groups 1A and 1B; zone groups 2A and 2B) was observed in the statistical characteristic of the 25% quantile (76%—Fig. 7a2—light blue sector of pie chart). Whereas the minimum value of the percentage share between maximum values of zone groups is 37% (Fig. 7b5) in the statistical characteristic of maximum slope gradients.

The highest determined ***value of the percentage share*** between ***minimum values*** of evaluated zone groups (1A and 1B; 2A and 2B) was recorded as 63% (14.2°) in the statistical characteristic of maximum slope gradients (Fig. 7b5—pink sector of pie chart). Conversely, the minimum value of the percentage share between zone groups (1A and 1B; 2A and 2B) is 24% in the statistical characteristics of the 25% quantile (0.7°) and 50% quantile (1.2°).

## Conclusion

The evaluated study has shown that the range of values in the box plot gradually increases from zones with less favourable physical–mechanical properties to zones with better physical–mechanical properties. Another finding of the study is the fact that soil masses have a lower range of box plot values of slope gradients than zones of rock and weathered rock masses with their eluvium. It is a well-known fact that soil masses have poorer physical–mechanical properties than rock and weathered rock masses. The study’s significant scientific value lies in the manifestation of this generally known fact into specific statistical values of slope gradients in the specific environment of engineering geological zones in the Czech Republic.

***Scientific Study 2 demonstrated*** an evident difference between the slope gradient values in engineering geological zones (Group EG zones 2A), where the mass consists of rocks and weathered rocks with their eluvium, and between zones where a soil mass is present (Group EG zones 2B). A similar trend was observed (***Scientific Study 1***) in similar groups of zones consisting of Quaternary sediments (Group EG zones 1A) and Pre-Quaternary sediments (Group EG zones 1B). The difference between these two studies in the Czech Republic represents zone Nk. In Study 1, it was part of the group of Pre-Quaternary zones 2A, whereas in the second evaluated Study 2, it was part of the opposite group (Group EG zones 1A), which was a group of engineering geological zones of soils. The reason was that, unlike other engineering geological zones in this group, it does not consist of rocks and weathered rocks but of soil, as there was no diagenetic consolidation of the sediment due to its younger age (Neogene), and also because there was not a sufficiently thick overburden to diagenetically transform it.

The most important finding is the demonstration of differences in minimum and maximum slope values of both evaluated groups of engineering geological zones the difference between zones 2A and 2B; the difference between 1A and 1B (difference between zones 2A and 2B; difference between 1A and 1B). It is evident that soil masses and rock and weathered rock masses must have different manifestations because they are mainly characterized by different physical–mechanical properties. This logically reflects in slope gradients, which were quantified in the Czech Republic through the study.

Soil masses (***Group EG zones 2B***) and rock and weathered rock masses (***Group EG zones 2A***) had a difference ***in minimums*** of 3.0° in the statistical characteristic of average slope gradient, representing a 41 percent share in the total sum of both evaluated differences (***Study 2***). Whereas the ***difference between maximums*** of both evaluated groups of zones is 4.4°, representing a 59 percent share. Therefore, the percentage difference between Group 2A and 2B is 18%.

When evaluating Study 1, ***the difference in minimums of average slope gradients*** in the group of pre-Quaternary (***Group EG zones 1A***) and Quaternary engineering-geological zones (***Group EG zones 1B***), the research found to be 1.7° (28% share in the total sum of both evaluated differences). Furthermore, it was found that the difference in ***maximum*** average values in Group 1A and 1B zones is 4.4° (72%). Thus, the percentage difference between Group 1A and 1B is 44%.

Thus, the difference in average slope gradients between minimums in both studies (1 and 2) is 1.3°, indicating that Study 2 is more objective from the perspective of engineering geology and geotechnics, as it further reflects the differences between soil and rock masses. Whereas Study 1 primarily has rocks and weathered rocks in the group of Pre-Quaternary engineering geological zones 1A but also a soil mass in zone Nk (Alternating sandy and gravely sediments).

When evaluating ***the difference in average slope gradients within individual groups of zones***, it is evident that a larger difference of 5.6° (57% share in the total sum of both differences) in slope values is observed in zones of rocks and weathered rocks with their eluvium (***Group EG zones 2A***) compared to soil zones (***Group EG zones 2B***) with 4.2° and a 43% share in the total sum of both differences. If we compare the same thing in the group of engineering geological zones ***1A*** (Pre-Quaternary zones) and ***1B*** (Quaternary zones), the Quaternary zones have the same value difference in slope gradient of 4.2° as in Group ***2B***, but the percentage share is lower at 38% (whereas Group 2B had a 43% share). However, a greater difference in slope gradient is observed in the group of zones 1A with 6.9° and 62%, unlike Group 2A, where the value was lower at 5.6° (57%).

Since all statistical characteristics of slope gradients in engineering geological zones in the Czech Republic have similar trends (except for extreme values of statistical characteristics of maximum slope gradients), the most relevant statistical characteristic of average slope gradient was used for quantification. Trends were similar for both investigated studies 1 and 2. Therefore, the trends were similar for both Study 1, with the evaluation of Pre-Quaternary engineering geological zone group (Group 1A) and Quaternary (Group 1B), and for Study 2, with the evaluation of zones of rocks and weathered rocks with their eluvium (Group 2A), and soil zones (Group 2B).

Soil masses have lower slope gradients unlike rock and weathered rock masses with their eluvium. This is entirely logical and corresponds to their material nature and the structure of the rock mass, which is reflected in physical–mechanical properties manifested on the surface in a single parameter, which is the evaluated slope gradient.

## Data Availability

All data generated or analysed during this study are included in this published article.
